# Predictive Analysis of Health/Physical Fitness in Health-Promoting Lifestyle of Adolescents

**DOI:** 10.3389/fpubh.2021.691669

**Published:** 2021-08-18

**Authors:** Hao Liu, Yiwen Liu, Bin Li

**Affiliations:** ^1^College of Physical Education, Southwest Medical University, Luzhou, China; ^2^College of Physical Education, North Sichuan Medical College, Nanchong, China

**Keywords:** health physical fitness, health promoting lifestyle, canonical correlation, predictive power, predictive analysis

## Abstract

**Objective:** Explore the correlation and predictive power of health physical fitness on health-promoting lifestyle of adolescents to provide an important reference for reform in middle school physical education classroom teaching.

**Methods:** Taking some junior and senior high school students in Nanchong City of Sichuan Province as survey objects, a stratified random sampling method was used to carry out a health/fitness test and conduct a questionnaire survey. SPSS17.0, amos 21.0, and other statistical analysis software were used to process the relevant data.

**Results:** (1) Male adolescents had 14.4% predictive power for their overall health-promoting lifestyle through the combined effects of cardiopulmonary endurance, muscle strength, and explosive strength, while female adolescents had 16.8% predictive power for their overall health-promoting lifestyle through the combined effects of cardiopulmonary endurance, flexibility, and body mass index (BMI). (2) Ten percent of the variation in the health-promoting lifestyle of men under 14 years old was caused by the combined effects of muscle endurance and muscle strength, while 14.4% of the variation in the health-promoting lifestyle of female adolescents was caused by the combined effects of muscle endurance, muscle strength, flexibility, and cardiopulmonary endurance. (3) Some 10.9 and 17.6% of the variation in health-promoting lifestyle of male and female adolescents aged between 14 and 17 was caused by the combined effects of cardiopulmonary endurance, muscle strength, and flexibility, respectively. (4) Some 20.7% of the variation in health promoting lifestyle of male adolescents aged 17 years or above was caused by cardiopulmonary endurance, explosive force, and muscle endurance, while 16.8% of the variation in health-promoting lifestyle of female adolescents was caused by the combined effect of cardiopulmonary endurance, BMI, and muscle endurance.

**Conclusion:** Health and physical fitness of adolescents can significantly predict their health-promoting lifestyle, and the predictive power and typical correlation between them are affected by demographic factors.

## Introduction

Fitness refers to the ability of body to adapt to life, sports, and environment, and consists of two parts: fitness for healthy body and fitness for competitive sports (1, 2). Healthy physical fitness is closely related to health, which means that the heart, lungs, vascular system, and muscle operation ability of individuals can exert effective function to complete daily activities without excessive fatigue, so they can have spare time to enjoy leisure and entertainment life and cope with emergencies (3–5). Healthy physical fitness includes five elements, namely, muscle strength, muscle endurance, flexibility, cardiopulmonary endurance, and body composition, which are related to the quality and ability of daily life or physical activities (6, 7). Health promotion theory points out that good health does not mean not to be ill, but means the overall state of personal happiness, balanced health behavior, and full lifestyle; that is to say, the environment of the individual has its unique way of action and has a positive or negative impact on personal health (8, 9). On the basis of Poder's theory, Sousa et al. (9) described health-promoting lifestyle as a multi-level spontaneous behavior and perception used by individuals to enhance or maintain happiness perception, self satisfaction, and self realization, and developed a health-promoting lifestyle scale with six dimensions: self realization, social support, health responsibility, exercise behavior, nutritional behavior, and stress management.

The relationship between the level of health physical fitness and health promoting lifestyle has long attracted the attention of scholars at home and abroad. Research shows that the five elements of health fitness are closely related to the quality and ability of human beings to engage in daily life or physical activities. With gradual decline in human organ and tissue function, the human body will gradually lose the ability to adapt to activities, thus causing diseases (10–14). Hammami et al. (15) pointed out that healthy physical fitness has positive functions for students, such as having sufficient physical strength to adapt to daily work, life, or reading; good health promotion and development level is conducive to the balanced development of all aspects of the individual, and provides a happy and lively lifestyle, so as to form good health habits. Baginska et al. (16) believe that having good health and being physically fit are the most basic conditions for teenagers to establish a good quality of life. Those who are physically fit are more energetic to complete their daily study, feel energetic, have spare time to enjoy extracurricular sports activities, and can easily cope with occasional physical discomfort. Huang and Qiu (17) found that healthy physical fitness of adolescents is closely related to their health and diseases, and it needs to be promoted through exercise. However, more empirical research is needed on the most effective way of exercise to improve their healthy physical fitness. Mooney et al. (18) pointed out in the revised model of health promoting behavior theory that personal characteristics and previous experience will directly or indirectly affect previous health promotion behavior, and that the influence degree of behavior will change the subsequent health behavior because of the target behavior of the individual; the influence of cognition and emotion of behavior characteristics will indirectly or directly affect the decision-making of health promotion behavior with cognitive factors and situational reactions. However, among the variables that restrict the health-promoting lifestyle mentioned by the above scholars, few scholars clearly point out whether the level of individual health fitness affects and predicts the health-promoting lifestyle.

To sum up, health/physical fitness is closely related to health-promoting lifestyle. However, lifestyle is a way of life or attitude for people to deal with their daily activities. It is all behaviors that a person can control, such as dangerous actions that affect personal health and arbitrary behaviors that causes harm to their health (9, 19). According to the available literature, few people in China have made a clear arrangement of the relationship between the two, and there is less exploration on whether health fitness can predict health-promoting lifestyle. Based on this, this study boldly put forward the following assumptions and tested them step by step: Hypothesis 1: there is a canonical correlation between adolescent health/physical fitness and health-promoting lifestyle, and this canonical correlation is affected by demographic variables. Hypothesis 2: The health/physical fitness of adolescents has significant predictive power on their overall health-promoting lifestyle, and this predictive power is affected by demographic variables.

## Objects and Methods

### Objects

Eight middle schools were selected by stratified random sampling (according to the students of junior high school and senior high school, four common schools and four key schools in each level). Then, six classes of students from each middle school were randomly selected as subjects of health fitness test and questionnaire survey.

Members of the research group made contact with the leaders of the teaching and research departments of physical education in the eight middle schools, and with the help of physical education teachers of the relevant teaching classes, completed the physical fitness test and questionnaire survey of all the students in 48 classes from April 1 to May 25, 2020.

A total of 1,854 students completed the test results and questionnaire. After reviewing the relevant test indicators and questionnaire filling, 39 samples were removed, and 1,815 valid samples were obtained, of which 56.1% were men and 43.9% were women; junior high school students accounted for 52.3%, senior high school students accounted for 47.7%; age distribution: <14 years old accounted for 36.3%, 14–17 years old accounted for 52.9%, and ≥17 years old accounted for 10.9; 59.8% of students participated in student associations, 40.2% of students did not participate in any student associations; BMI was too light accounted for 29.2, 51.2, 19.6% of overweight (see Table 1).

**Table 1:** Descriptive statistical analysis of personal background information of respondents (*N* = 1815).

	**Gender**		**Grade**		**Age**		
	**Male**	**Female**	**Junior school student**	**Senior high school student**	**≤14**	**14–17**	**≥17**
Ordinary middle school	526 (60.7%)	341 (39.3%)	445 (51.3%)	422 (48.7%)	323 (37.3%)	445 (51.3%)	99 (11.4%)
Key middle schools	493 (52.0%)	455 (48.0%)	505 (53.3%)	443 (46.7%)	335 (35.3%)	515 (54.3%)	98 (10.3%)
Total	1,019 (56.1%)	796 (43.9%)	950 (52.3%)	865 (47.7%)	658 (36.3%)	960 (52.9%)	197 (10.9%)
Test	X^2^ = 13.81; *P* = 0.000 <0.001		X^2^ = 0.686; *P* = 0.408 > 0.05		X^2^ = 1.72; *P* = 0.424 > 0.05		
	**Participate in student associations**		**Family structure**		**Body mass index (BMI)**		
	**Yes**	**No**	**Two-parent family**	**Other family**	**Too light**	**Standard**	**Overweight**
Ordinary middle school	590(68.1%)	277(31.9%	707(81.5%)	160(18.5%)	266(30.7%)	429(49.5%)	172(19.8%)
Key middle schools	496(52.3%)	452(47.7%)	714(75.3%)	234(24.7%)	264(27.8%)	501(52.8%)	183(19.3%)
Total	1086(59.8%)	729(40.2%)	1421(78.3%)	394(21.7%)	530(29.2%)	930(51.2%)	355(19.6%)
Test	X^2^ = 46.62; *P* = 0.000 <0.001		X^2^ = 10.34; *P* = 0.001 <0.01		X^2^ = 2.31; *P* = 0.315 > 0.05		

### Method

#### Measuring Method

##### Selection of Health Fitness Index

All measurement tools are the instruments and equipment uniformly stipulated by the national physique measurement of China.

1) Height and weight: the tool is height-weight meter.
2) Body mass index (BMI): to evaluate body fat content, the formula is: BMI = weight (kg)/height (M)^2^.
3) Sitting posture flexion (flexibility): the value is measured with a sitting posture flexion detector (unit: cm)
4) Thirty-second sit up (muscle strength): 30 s knee flexion sit up and the test is completed in 30 s.
5) One-minute sit up (muscle endurance): 60 s knee flexion sit up, record the number of times after 1 min test.
6) Standing long jump (explosive force): standing long jump pad (special pad for college entrance examination of physical education, unit: cm).
7) Cardiopulmonary endurance (female 800 m/male 1,500 m running, unit: s): reflects cardiopulmonary function or aerobic fitness level.

#### Questionnaire Survey Method

##### Questionnaire Structure

In this study, we used the Chinese short form of “health-promoting lifestyle” developed by Chung et al. (20). There were 20 items in the scale, and included five dimensions, namely sports behavior, nutritional behavior, self realization, interpersonal support, and health responsibility. Each subscale had four items. The answers were “always like this” 4 points, “often like this” 3 points, “occasionally like this” 2 points, and “never like this” 1 point. The higher the score, the better the performance of health-promotion behavior

The validity and reliability of the scale in Southwest China were tested by pre-test before the formal questionnaire was given. Two classes of students were randomly selected from each of the two middle schools for investigation, and prediction was completed from January 1 to February 1, 2020.

##### Reliability and Validity Test

Table 2 shows that according to the predicted data of the health-promoting lifestyle scale, the number of common factors extracted by the principal axis method is 5, of which KMO = 0.89 and Bartlett spherical test values are significant (*P* < 0.001), indicating that the scale is suitable for factor analysis. The cumulative explained variance of the five common factors is 79.54%. After rotation, the relationship characteristics are obvious, and the five common factors contain 20 items. The four items of the first common factor are mainly related to social support variables related to adolescent health. The four items of the second common factor are mainly related to the attention paid by adolescents to their own health, the responsibility they accept, and the need to seek professional assistance. The four items of the third common factor are mainly related to a positive perception of life, goal perception, and optimistic perception of life. The fourth common factor is mainly related to social support variables related to adolescent health, and its four items are mainly related to daily diet and food choices of teenagers. The four items of the fifth common factor are mainly related to sports and leisure activities of teenagers inside and outside school. The internal consistency test results of the five common factors showed that Cronbach's α coefficients were 0.87, 0.81, 0.84, 0.85, and 0.79, respectively, indicating that the health-promoting lifestyle scale has high reliability and validity.

**Table 2:** Validity and reliability of the health-promoting lifestyle scale.

**Dimension naming**	**Item**	**KMO value and sphericity test Bartlett**	**Eigen value**	**Explained variance%**	**Variance% of progressive interpretation**	**Cronbach α coefficient**	**Score**	**Ranking**
Interpersonal support	4		14.06	28.12	28.12	0.87	3.11 ± 0.69	1
Health responsibility	4	KMO = 0.89	9.38	18.76	46.88	0.81	2.68 ± 0.71	2
Self-realization	4	*P* = 0.000 <0.001	7.65	15.30	62.18	0.84	2.51 ± 0.82	3
Nutritional behavior	4		5.14	10.28	72.46	0.85	2.37 ± 0.53	4
Sports behavior	4		3.54	7.08	79.54	0.79	2.09 ± 0.72	5

### Data Processing

SPSS for Windows 17.0 and amos 21.0 data processing software were used. The specific methods mainly include mean comparison, analysis of variance, regression analysis, and canonical correlation analysis. The statistical significance level of all variables was set as α = 0.05.

## Results

### Correlation and Predictive Power of Health/Physical Fitness on Health-Promoting Lifestyle in Adolescents of Different Genders

#### Analysis of Gender Characteristics of Relevance

**Figure 1:**
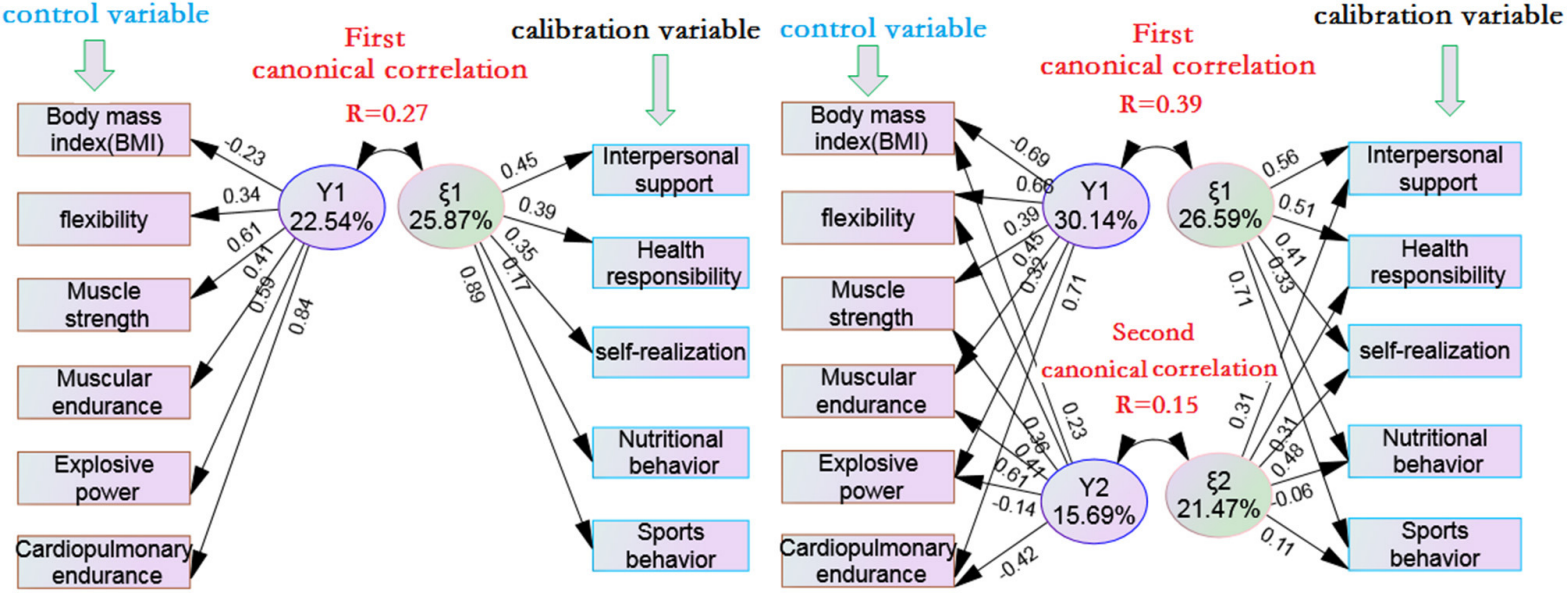
Canonical correlation between health physical fitness and health promoting lifestyle in adolescents of different genders.

Figure 1 shows the following:

1) There was only one group of typical correlation between health/physical fitness and health-promoting lifestyle in male adolescents, with a canonical correlation coefficient of *r* = 0.27 (*p* < 0.001), indicating that the canonical factor Y_1_ of the control variable could explain 7.3% (*R*^2^ = 0.27 × 0.27 = 0.073) of the total variance of the canonical factor ξ_1_ of the calibration variable. From the percentage of extracted variance, the typical factor Y_1_ can explain about 22.54% of the variance of six control variables on average, while the typical correlation factor ξ_1_ can explain about 25.87% of the variance of five calibration variables on average. The canonical structure coefficient showed that Y_1_ was positively correlated with cardiopulmonary endurance (*r* = 0.84^**^), muscle strength (*r* = 0.61^**^), and explosive power (*r* = 0.59^**^), while ξ_1_ was positively correlated with exercise behavior (*r* = 0.89^**^) in calibration variables, and positive correlation between middle and low degree with interpersonal support (*r* = 0.45^**^) and health responsibility (*r* = 0.39^*^). It can be seen that the health/physical fitness of male adolescents is mainly through cardiopulmonary endurance, muscle strength, and explosive force, which has a high correlation with sports behavior in health-promoting lifestyle.
2) There are two groups of typical significant correlation between the health/physical fitness and health-promoting lifestyle of female adolescents, of which canonical correlation coefficients were *r* = 0.39^**^ (*p* < 0.001) and *R* = 0.15^*^(*p* < 0.05), respectively. Obviously, the explanatory amount of the first group of canonical correlation is relatively large. The canonical factor Y_1_ of the control variable can explain 15.2% of the total variation of the canonical factor ξ_1_ of the calibration variable (*R*^2^ = 0.39 × 0.39 = 0.152), while the canonical factor Y_1_ of the second group of canonical correlation can only explain 2.25% of the total variation of the canonical factor ξ_1_ of the calibration variable (*R*^2^ = 0.15 × 0.15 = 0.0225). From the percentage of extracted variance, the first typical factor Y_1_ can explain about 30.14% of the variance of the six control variables on average, while the typical correlation factor ξ_1_ can explain about 26.59% of the variance of the five calibration variables on average. The second canonical factor Y_1_ can explain about 15.69% of the variance of the six control variables, while the canonical correlation factor ξ_1_ can explain about 21.47% of the variance of the five calibration variables. The canonical structure coefficient showed that in the first canonical correlation, Y_1_ was moderately positively correlated with cardiopulmonary endurance (*r* = 0.71^**^), flexibility (*r* = 0.66^**^), and moderately negatively correlated with BMI (*r* = −0.69^**^), while ξ_1_ was moderately positively correlated with exercise behavior (*r* = 0.71^**^), interpersonal support (*r* = 0.56 ^**^), and health responsibility (*r* = 0.51^**^). In the second canonical correlation, Y_2_ had a moderate positive correlation with muscle endurance (*r* = 0.61^**^), while ξ_2_ had a low positive correlation with self realization (*r* = 0.48^*^). Based on the canonical correlation between the two groups, it shows that the health/physical fitness of female adolescents is positively related to exercise behavior, interpersonal support, and health responsibility in health-promoting lifestyle through cardiopulmonary endurance, flexibility, BMI, and muscle endurance.

#### Gender Characteristics of Predictive Power

Table 3 shows the following:

1) The variables that were significantly introduced into the regression equation were cardiopulmonary endurance, explosive force, and muscle strength, and multiple correlation coefficient *r* = 0.38^**^ (*R*^2^ = 0.38 × 0.38 = 0.144). It can be considered that 14.4% of the variation in the health-promoting lifestyle of the boys was caused by the combined effect of cardiopulmonary endurance, explosive force, and muscle strength. The standardized coefficients β of the three variables were 0.243^**^, 0.187^**^, and 0.161^**^, respectively, and the increase in the explanatory volume ▲*R*^2^ showed that the contribution rates of cardiopulmonary endurance, explosive force, and muscle strength to health-promoting lifestyle changes were 6.3, 4.6, and 3.5%, respectively. The results showed that the predictive effect of cardiopulmonary endurance (i.e., “3 min step up”) was the best, followed by explosive force (i.e., “standing long jump”), and the predictive effect of muscle strength (i.e., “30 s sit up”) was the least.
2) There are four individual fitness indexes significantly introduced into the regression equation: cardiopulmonary endurance, BMI, flexibility (“sitting and flexing”), and muscle endurance; multiple correlation coefficient of regression equation *R* = 0.41^**^ (*R*^2^ = 0.41 × 0.41 = 0.168), which can be considered as cardiopulmonary endurance. BMI, flexibility, and muscle endurance could jointly predict 16.8% of changes in health-promoting lifestyle. The standardized coefficients β of the four variables were 0.271^**^, 0.225^**^, 0.207^**^, and 0.116^**^, respectively. The increase in ▲*R*^2^ showed that cardiopulmonary endurance, BMI, flexibility, and muscle endurance contributed 7.3, 4.3, 3, and 1.6%, respectively, to health-promoting lifestyle changes. The results showed that the predictive power of cardiopulmonary endurance was the best, followed by BMI, flexibility, and muscle endurance.

**Table 3:** Statistical table of multiple stepwise regression analyses of health and physical fitness of men and women on their overall health promoting lifestyle.

**Variable name**	**Health promoting lifestyle**			**Health promoting lifestyle**			
	**(Male)**			**(Female)**			
	**Equation 1(β)**	**Equation 2(β)**	**Equation 3(β)**	**Equation 1(β)**	**Equation 2(β)**	**Equation 3(β)**	**Equation 4(β)**
Body mass index BMI					0.225^**^	0.225^**^	0.225^**^
flexibility						0.207^**^	0.207^**^
Muscle strength			0.161^**^				
Muscular endurance							0.116^**^
Explosive power		0.187^**^	0.187^**^				
Cardiopulmonary endurance	0.243^**^	0.243^**^	0.243^**^	0.271^**^	0.271^**^	0.271^**^	0.271^**^
Complex correlation coefficient R	0.25	0.33	0.38	0.27	0.34	0.39	0.41
Determination coefficient *R*^2^	0.063	0.109	0.144	0.073	0.116	0.152	0.168
Interpretation increment ▲**R**^2^	0.063	0.046	0.035	0.073	0.043	0.036	0.016
Statistics, F	12.14^***^	9.47^***^	7.15^**^	14.06^***^	8.69^***^	7.32^**^	5.16^*^

* *P < 0.05*;

** *P < 0.01*;

*** *P < 0.001*.

### Correlation and Predictive Power of Health Physical Fitness on Health-Promoting Lifestyle in Adolescents of Different Ages

#### Analysis of Age Characteristics of Relevance

**Figure 2:**
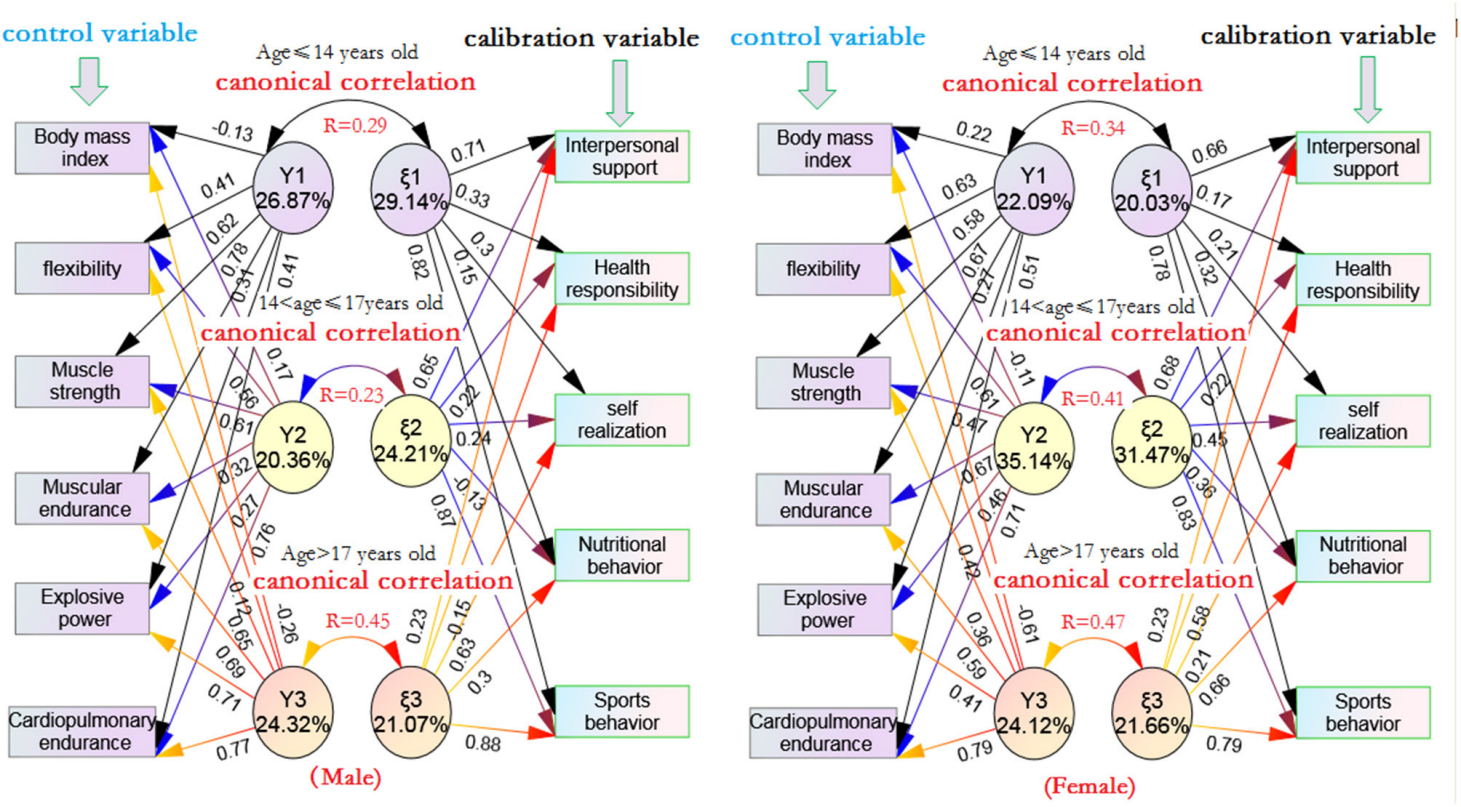
Typical correlation characteristics between health physical fitness and health promoting life of adolescents of different ages.

Figure 2 shows the following:

1) There was a significant canonical correlation between health/physical fitness and health-promoting lifestyle in boys and girls under 14 years old. The canonical correlation coefficients R were 0.29^**^ and 0.34^**^, respectively. The canonical factor Y_1_ of the male and female control variables could explain 8.41% (*R*^2^ = 0.29 × 0.29 = 0.0841) and 11.56% (*R*^2^ = 0.34 × 0.34 = 0.1156) of the total variance of canonical correlation factor ξ_1_ of calibration variables, respectively. From the percentage of extracted variance, the first typical factor Y_1_ of male and female could explain 26.87 and 22.09% of the average variance of the six control variables, respectively, while the typical correlation factor ξ_1_ of male and female could explain 29.14 and 20.03%, respectively, of the average variance of the five calibration variables. From the perspective of typical structure coefficient, male Y_1_ was highly positively correlated with muscle endurance (0.78^**^) and muscle strength (0.62^**^), while female Y_1_ was moderately positively correlated with cardiopulmonary endurance (0.51^**^), muscle strength (0.58^*^), muscle endurance (0.67^**^), and flexibility (0.56^*^); male calibration variable extraction ξ_1_ was moderately positively correlated with interpersonal support (0.71^**^) and exercise behavior (0.82^**^). Similarly, the women also had a moderate and high positive correlation with sports behavior (0.78^**^) and interpersonal support (0.66^**^). It can be seen that boys aged ≤ 14 years old have a positive correlation with the exercise behavior and interpersonal support of health-promoting lifestyle mainly through muscle strength and muscle endurance, while girls have a high positive correlation with the exercise behavior and interpersonal support of health-promoting lifestyle through cardiopulmonary endurance, muscle strength, muscle endurance, and flexibility.
2) There was a significant canonical correlation between healthy physical fitness and health-promoting lifestyle in men and women 14 < age ≤ 17 years old, and the canonical correlation coefficients were 0.23^**^ and 0.41^**^, respectively; that is to say, the canonical factor Y_2_ of male and female control variables could explain 5.29% (*R*^2^ = 0.23 × 0.23 = 0.0529) and 16.81% (*R*^2^ = 0.41 × 0.41 = 0.1681), respectively, of the total variance of the canonical correlation factor ξ_2_ of calibration variables. From the percentage of extracted variance, male and female Y_2_ could explain about 20.36 and 35.14%, respectively, of the variance of the six control variables, while male and female ξ_2_ could explain about 24.21 and 31.47%, respectively, of the variance of the five calibration variables. The canonical structure coefficient showed that male Y_2_ had a moderate correlation with cardiopulmonary endurance (0.76^**^), muscle strength (0.61^**^), and flexibility (0.56^*^), and that its calibration variable ξ_2_ had a moderate positive correlation with sports behavior (0.87^**^) and interpersonal support (0.65^**^). Female Y_2_ had a moderate positive correlation with cardiopulmonary endurance (0.71^**^), muscle endurance (0.67^**^), and flexibility (0.61^**^), while the extraction of calibration variables ξ_2_ had a moderate high positive correlation with sports behavior (0.83^**^) and interpersonal support (0.68^**^). It can be seen that boys aged 14–17 have a positive correlation with exercise behavior and interpersonal support in health-promoting lifestyle mainly through cardiopulmonary endurance, muscle strength, and flexibility, while girls have a positive correlation with exercise behavior and interpersonal support in health-promoting lifestyle mainly through cardiopulmonary endurance, muscle strength, and flexibility.
3) There was only one group of typical significant correlation between health/physical fitness and health-promoting lifestyle of male and female students over 17 years old, and the canonical correlation coefficient *r* was 0.45^**^ and 0.47^**^, respectively; that is to say, the typical factor Y_3_ of male and female control variables could explain 20.25% (*R*^2^ = 0.45 × 0.45 = 0.2025) and 22.09% (*R*^2^ = 0.47 × 0.47 = 0.2209), respectively, of the total variance of the canonical correlation factor ξ_3_ of calibration variables. From the percentage of extracted variation, the male typical factor Y_3_ can explain about 24.32% of the six control variables on average, and its calibration variable extraction amount ξ_3_ can explain about 21.07% of the five calibration variables on average. The female typical factor Y_3_ can explain about 21.07% of the six control variables on average, and its calibration variable extraction amount ξ_3_ can explain about 21.66% of the five calibration variables on average. The canonical structure coefficient showed that male Y_3_ was positively correlated with cardiopulmonary endurance (0.77^**^), muscle endurance (0.65^**^), and explosive power (0.69^**^), while the extraction of calibration variable ξ_3_ was positively correlated with exercise behavior (0.88^**^) and self realization (0.63^**^). Female Y_3_ had a high correlation with cardiopulmonary endurance (0.79^**^), muscle endurance (0.59^*^), and body mass index (−0.61^**^), and its calibration variable extraction amount ξ_3_ had a high positive correlation with exercise behavior (0.79^**^), nutritional behavior (0.66^**^), and health responsibility (0.58^*^). It can be seen that the health/physical fitness of male adolescents over 17 years old is mainly through cardiopulmonary endurance and muscle endurance, which has a high positive correlation with the exercise behavior and self realization of health-promoting lifestyle, while the female adolescents are positively correlated with the exercise behavior, nutrition behavior, and health responsibility of health-promoting lifestyle through cardiopulmonary endurance, muscle endurance, and BMI.

#### Age Characteristics of Predictive Power

Table 4 shows the following:

1) In the prediction of the overall health-promoting lifestyle by the health/physical fitness of male adolescents aged ≤ 14 years old, the multiple multiple correlation coefficient is *R* = 0.31^**^, which indicates that 10% (*R*^2^ = 0.31 × 0.31 = 0.1) of the variation in the health-promoting lifestyle of boys in this age group can be caused by the combined effect of muscle endurance and muscle strength; the standardized coefficients β of the two variables were 0.216^**^ and 0.196^**^, which indicated that the influence of “muscle endurance” on health-promoting lifestyle was close to that of “muscle strength.” In terms of the size of interpretation increment ▲*R*^2^, the contribution rate of muscle endurance and muscle strength to health-promoting lifestyle was 3.4 and 1.8%, respectively. Among the male adolescents aged 14 < age ≤ 17 years old, the indexes of physical fitness with significant regression equation were cardiopulmonary endurance, muscle strength, and flexibility; the multiple multiple regression coefficient r was *R* = 0.33^**^, which indicated that 10.9% (*R*^2^ = 0.33 × 0.33 = 0.1089) of the changes in health-promoting lifestyle of boys were caused by the combined effects of cardiopulmonary endurance, muscle strength, and flexibility. For the male adolescents 17 years old, the results showed that 20.3% (*R*^2^ = 0.45 × 0.45 = 0.2025) of the variation in lifestyle was caused by cardiopulmonary endurance, explosive force, and muscle endurance. Judging from the size of interpretation increment ▲*R*^2^, cardiopulmonary endurance has no effect on the health-promoting lifestyle of men under 14 years old; the contribution rates of cardiopulmonary endurance, muscle strength, and flexibility to health-promoting lifestyle changes were 5.3, 3.1, and 2.5%, respectively, in boys aged 14–17 years, while the contribution rates of cardiopulmonary endurance, muscle strength, and flexibility to health-promoting lifestyle changes were 8.4, 5.3, and 6.6%, respectively, in boys aged over 17 years.
2) In the prediction of the overall health-promoting lifestyle by the health physical fitness of female adolescents aged ≤ 14 years old, muscle endurance, muscle strength, flexibility, and cardiopulmonary endurance were significantly introduced into the regression equation, and the multiple multiple correlation coefficient was *R* = 0.38^**^, indicating that 14.4% (*R*^2^ = 0.38 × 0.38 = 0.1444) of the changes in the health-promoting lifestyle of female adolescents under 14 years old were caused by the combined effects of muscle endurance, muscle strength, flexibility, and cardiopulmonary endurance; From the explanation increment ▲*R*^2^, the contribution rates of muscle endurance and muscle strength, flexibility, and cardiopulmonary endurance to the change in health-promoting lifestyle were 8.4, 3.2, and 2.8, respectively, which indicated that the combined effect of muscle endurance and muscle strength had the greatest influence on health-promoting lifestyle, followed by that of flexibility and cardiopulmonary endurance. Among the female adolescents aged 14–17 years old, cardiopulmonary endurance, muscle endurance, and flexibility were significantly introduced into the regression equation, and the corresponding multiple regression coefficient r was *R* = 0.42^**^, which indicated that 17.6% (*R*^2^ = 0.42 × 0.42 = 0.1764) of the health-promoting lifestyle changes in the female adolescents aged 14 < year ≤ 17 were caused by the combined effects of cardiopulmonary endurance, muscle endurance, and flexibility. For the adolescent girls over 17 years old, the indexes introduced into the regression equation were cardiopulmonary endurance, BMI, and muscle endurance, and the corresponding multiple regression coefficient r was *R* = 0.41^**^, which indicated that 16.8% (*R*^2^ = 0.41 × 0.41 = 0.1681) of the health-promoting lifestyle changes in the female adolescents aged >17years old were caused by the combined effects of cardiopulmonary endurance, BMI, and muscle endurance. In terms of explaining the magnitude of increment ▲*R*^2^, the contribution rates of cardiopulmonary endurance, muscle endurance, and flexibility to health-promoting lifestyle changes were 7.8, 4.5, and 5.3%, respectively, in the girls aged 14–17 years, while the contribution rates of cardiopulmonary endurance, muscle endurance, and BMI to health-promoting lifestyle changes were 7.8, 4.5, and 4.5%, respectively, in the girls aged over 17 years.

**Table 4:** Multiple stepwise regression analysis statistics of health and physical fitness of adolescents of different ages on their overall health-promoting lifestyle.

**Male model →**	**≤14 years old**			**14 < age≤17**			**>17 years old**		
	**Equation 1(β)**	**Equation 2(β)**	**Equation 3(β)**	**Equation 1(β)**	**Equation 2(β)**	**Equation 3(β)**	**Equation 1(β)**	**Equation 2(β)**	**Equation 3(β)**
Body mass index BMI									
Flexibility						0.127^**^			
Muscle strength			0.196^*^		0.189^**^	0.186^**^			
Muscular endurance	0.217^**^	0.217^**^	0.216^**^						0.189^**^
Explosive power		0.262^**^	0.262^**^					0.216^**^	0.216^**^
Cardiopulmonary endurance				0.241^**^	0.241^**^	0.237^**^	0.302^**^	0.302^**^	0.302^**^
Complex correlation coefficient R	0.21^**^	0.28^**^	0.31^**^	0.23^**^	0.29^**^	0.33^**^	0.29^**^	0.37^**^	0.45^**^
Determination coefficient *R*^2^	0.044	0.078	0.096	0.053	0.084	0.109	0.084	0.137	0.203
Interpretation increment ▲**R**^2^	0.044	0.034	0.018	0.053	0.031	0.025	0.084	0.053	0.066
Statistics, F	10.26^**^	9.24^**^	11.02^**^	13.05^**^	9.37^**^	11.36^**^	10.85^**^	12.78^**^	17.023^**^
**Female model →**	**≤14 years old**			**14 < age ≤ 17**			**>17 years old**		
Body mass index BMI								−0.206^**^	-0.206^**^
Flexibility		0.181^**^	0.181^**^			0.192^**^			
Muscle strength	0.205^**^	0.201^**^	0.201^**^						
Muscular endurance	0.229^**^	0.224^**^	0.224^**^		0.246^**^	0.246^**^			0.178^**^
Explosive power									
Cardiopulmonary endurance			0.158^**^	0.307^**^	0.307^**^	0.307^**^	0.254^**^	0.254^**^	0.254^**^
Complex correlation coefficient R	0.29^**^	0.34^**^	0.38^**^	0.28^**^	0.35^**^	0.42^**^	0.28^**^	0.35^**^	0.41^**^
Determination coefficient *R*^2^	0.084	0.116	0.144	0.078	0.123	0.176	0.078	0.123	0.168
Interpretation increment ▲**R**^2^	0.084	0.032	0.028	0.078	0.045	0.053	0.078	0.045	0.045
Statistics, F	13.28^**^	11.21^**^	9.27^**^	9.06^**^	13.29^**^	8.58^**^	14.27^**^	12.77^**^	10.66^**^

* *P < 0.05*;

** *P < 0.01*.

## Discussion

### Gender Characteristics of Adolescent Health/Physical Fitness and Health-Promoting Lifestyle

1) In this study, we found that the number of effective standard variables in the first canonical correlation factors of male and female adolescents was almost the same (25.87 vs. 26.59%), indicating that the effect of male and female health physical fitness on their overall health-promoting lifestyle was very close. However, from the contribution of the amount of extraction, interpersonal support (regression standardized coefficient 0.45^*^) and health responsibility (regression standardized coefficient 0.39^*^) for male students were significantly lower than those for female students (regression standardized coefficient 0.56^*^and 0.51^*^, respectively). The contribution of sports behavior to the overall health promotion of male students was significantly better than that of female students (regression standardized coefficient 0.89^**^ vs. 0.71^**^). The reason for this difference may come from the gender role orientation given by society. Men are considered to be independent in personality, lively, active, and energetic in behavior, while women are more likely to be cared for and used to seeking support from others. In behavior, they are considered to be quiet and dignified, and even in a physical education class, some teacher requirements and expectations for the physical fitness of girls are automatically reduced. The long-term accumulation of such rigid thinking reduces women's interest and learning in healthy physical fitness (21–23). Sidani et al. (24) believe that women emphasize personal interpersonal relationships in terms of interpersonal support, and they usually solve problems in an interpersonal and intimate way. Szlezak et al. (25) found that girls have a more active and positive attitude toward help-seeking than men when they encounter behavioral distress, while men are less willing to disclose their thoughts and are more passive in interpersonal relationships. Therefore, the findings of this study are consistent with the results of previous ones.
2) From the perspective of the predictive power of the health/physical fitness of men on the overall health-promoting lifestyle, cardiopulmonary endurance, explosive force, and muscle strength jointly affect the exercise behavior, interpersonal support, and health responsibility in health-promoting lifestyle, and have significant predictive power on the overall health-promoting lifestyle. The contribution rate (▲*R*^2^) confirms that cardiopulmonary endurance plays the most important role. Compared with men, female students significantly affected the exercise behavior, interpersonal support, and health responsibility in health-promoting lifestyle through cardiopulmonary endurance, BMI, flexibility, and muscle endurance. These findings enlighten us that in the content arrangement of middle school physical education, we should consider the gender differences between men and women. For female teenagers, we should consider adding bounce and agility training to improve their flexibility, and strengthen endurance training to improve their cardiopulmonary function. For male teenagers, we should pay attention to muscle strength training, especially explosive strength training. In addition, this study also found that the attention of women to BMI was significantly higher than that of men. The first canonical correlation showed that BMI had almost no contribution to control variable extraction for men (*r* = −0.23, not significant), but had a significant contribution to the index for women, and (*r* = −0.69^*^), which showed a significant inverse relationship between health-promoting lifestyle and BMI. In other words, poor lifestyle shows a higher BMI, which can explain why in the first canonical correlation, health responsibility for women is significantly higher than that for men. At the same time, this result also supports the research findings of some scholars (26–28).

### On the Age Characteristics of Health/Physical Fitness of Adolescents and Health-Promoting Lifestyle

1) This study found that men over 17 years old mainly change their health-promoting lifestyle through cardiopulmonary endurance, explosive power, and muscle endurance, while women mainly rely on cardiopulmonary endurance, BMI, and muscle endurance. From the overall contribution of health-promoting lifestyle, both men and women at this age mainly rely on interpersonal support and sports behavior. This finding implies that the contents of health/physical fitness training can be different between male and female adolescents aged above 17, but that they can have the same effect on their health-promoting lifestyle, which is an interesting finding. On the other hand, from the perspective of the predictive power of health/physical fitness on the overall health-promoting lifestyle, significant variables of the regression equation, such as cardiopulmonary endurance, explosive power, and muscle endurance, can jointly predict 20.7% of the variance of health-promoting lifestyle for boys over 17 years old, while significant variables of the regression equation, such as cardiopulmonary endurance, BMI, and muscle endurance, can significantly predict the 16.8% variance in health-promoting lifestyle of female students. According to relevant research reports (29, 30), the better the explosive force, the faster the movement and greater power output. This seems to suggest that in the process of middle school physical education teaching, for boys over 17 years old, we should strengthen explosive force training and pay special attention to strength training in the core strength area, so as to improve stride length and stride frequency, which is beneficial to promote their healthy lifestyle and improve their health. For female students, more attention should be paid to cardiopulmonary endurance and BMI control.
2) This study found that male adolescents aged 14 < and ≤ 17 years mainly affect their overall health-promotion form through the combined effects of cardiopulmonary endurance, muscle strength, and flexibility, while female adolescents mainly rely on cardiopulmonary endurance, muscle endurance, and flexibility. From the overall health-promotion form extraction, the main contribution indicators of male and female are sports behavior and interpersonal support, which are the same as those of aged ≤ 14 years. This finding shows that, with the growth of age, male and female students in the teaching process of public physical education should pay attention to explosive force to strengthen cardiopulmonary endurance, while female students should pay attention to muscle strength for muscle endurance. On the other hand, from the perspective of the predictive power of health/fitness on the overall health-promoting lifestyle, the combined effects of cardiopulmonary endurance, muscle strength, and flexibility can significantly predict 10.9% of the variance in health-promoting lifestyle for men aged 14 <17 years; while the combined effects of cardiopulmonary endurance, muscle strength, and flexibility can significantly predict 10.2% of the variance in health-promoting lifestyle for women. It is particularly noteworthy that the regression equation highlights the role of flexibility in both male and female students. According to relevant research reports (Rodrigo et al., 2018), flexibility training can relieve daily muscle tension, pain, and soreness, increase movement fluency in daily life, and has positive significance for maintaining body balance, relaxation, and spiritual peace. Therefore, middle school public physical education should implement more soft exercises and cooperate with inhalation and exhalation abdominal breathing training. In addition, for women of this age group, while strengthening cardiopulmonary endurance training, muscle endurance training should also be emphasized. Ito et al. (31) reported that female students with poor muscle endurance or decreased critical moment are prone to injury or accidents, especially back injury. Therefore, for women of this age, more abdominal muscle endurance training should be carried out in the arrangement of public physical education courses.
3) This study found that men under 14 years old mainly affect their overall health-promotion form through the combined effect of cardiopulmonary endurance and muscle endurance, while women mainly rely on cardiopulmonary endurance, muscle endurance, and BMI. From the overall health-promotion form extraction, men mainly rely on sports behavior and self realization, while women mainly rely on sports behavior, nutrition behavior, and health responsibility. From the perspective of the predictive power of health/physical fitness on the overall health-promoting lifestyle, the combined effects of cardiopulmonary endurance, muscle endurance, and BMI could significantly predict 13.7% of the variance in health-promoting lifestyle of men over 17 years old, while the combined effects of cardiopulmonary endurance, muscle endurance, and BMI could significantly predict 14.4% of the variance in health-promoting lifestyle of women. Obviously, with the increase in age, the predictive power of men and women increased significantly. These findings remind us that for boys and girls over 17 years old, if they want to have a better health-promoting lifestyle, the emphasis of health fitness training should be on cardiopulmonary endurance and muscle endurance. At the same time, women should also attach great importance to BMI.

## Conclusion

1) The correlation and predictive power of health physical fitness on health-promoting lifestyle are affected by gender factors, which shows that men rely on the combined effects of cardiopulmonary endurance, muscle strength, and explosive strength, while women rely on the combined effects of cardiopulmonary endurance, flexibility, and BMI, which are, respectively, associated with sports behavior and interpersonal support in health-promoting lifestyle. The predictive power of women's physical fitness on the overall health promoting lifestyle was significantly higher than that of men.
2) The typical relevance and predictive power of adolescent health fitness to health promoting lifestyle are affected by age factors, which shows that low age boys mainly through cardiopulmonary endurance and explosive power, while high age men mainly through the combined effect of cardiopulmonary endurance and muscle endurance have positive correlation with health-promoting lifestyle respectively, but there was a significant age difference in the predictive power of the combined effect on the overall health-promoting lifestyle. Low age female students mainly through cardiopulmonary endurance, explosive force, muscle endurance, and flexibility, while high age female students mainly through cardiopulmonary endurance, muscle endurance, and BMI and other joint effects on health-promoting lifestyle, but this joint effect on the overall health-promoting lifestyle has no age difference.

## Data Availability Statement

The original contributions presented in the study are included in the article/supplementary material, further inquiries can be directed to the corresponding author/s.

## Ethics Statement

The studies involving human participants were reviewed and approved by Ethics Committee of College of physical education, Southwest University. Written informed consent to participate in this study was provided by the participants' legal guardian/next of kin.

## Author Contributions

HL and BL are mainly responsible for the research design and questionnaire survey. YL was responsible for the establishment of the database and statistical analysis of the data, and the writing of the paper is mainly completed by HL. All authors contributed to the article and approved the submitted version.

## Conflict of Interest

The authors declare that the research was conducted in the absence of any commercial or financial relationships that could be construed as a potential conflict of interest.

## Publisher's Note

All claims expressed in this article are solely those of the authors and do not necessarily represent those of their affiliated organizations, or those of the publisher, the editors and the reviewers. Any product that may be evaluated in this article, or claim that may be made by its manufacturer, is not guaranteed or endorsed by the publisher.
